# Doctors Are Soldiers

**Published:** 1918-09

**Authors:** 


### Doctors Are Soldiers.

There is an idea that doctors and nurses have safe places in the service and that they are seldom subjected to unusual dangers. The first United States soldier to be killed in the war and the first to be wounded were both in the medical corps. This fact must have impressed the American people with the serious risks which are taken by army doctors. Before we entered the war the bravery and sacrifice of medical officers at the front had probably been too little appreciated. As a matter of fact, the officers and enlisted men in the medical corps are the only soldiers who go into no man's land with no defense whatever. The tireless work on the battlefield of the stretcher bearers, all of whom are in the medical corps, is full of danger.

